# Aqua­bis(isonicotinamide-κ*N* ^1^)bis­(4-methyl­benzoato)-κ*O*;κ^2^ *O*,*O*′-cadmium(II) monohydrate

**DOI:** 10.1107/S1600536810008366

**Published:** 2010-03-13

**Authors:** Hacali Necefoğlu, Efdal Çimen, Barış Tercan, Yasemin Süzen, Tuncer Hökelek

**Affiliations:** a Department of Chemistry, Kafkas University, 36100 Kars, Turkey; b Department of Physics, Karabük University, 78050 Karabük, Turkey; c Department of Chemistry, Faculty of Science, Anadolu University, 26470 Yenibağlar, Eskişehir, Turkey; d Department of Physics, Hacettepe University, 06800 Beytepe, Ankara, Turkey

## Abstract

In the crystal structure of the title compound, [Cd(C_8_H_7_O_2_)_2_(C_6_H_6_N_2_O)_2_(H_2_O)]·H_2_O, the Cd^II^ cation is coordinated by two 4-methyl­benzoate (PMB) anions, two isonicotinamide (INA) ligands and one water mol­ecule in a distorted octa­hedral CdN_2_O_4_ geometry. One of PMB ions acts as a bidentate ligand while the other and the two INA are monodentate ligands. An O—H⋯O hydrogen bond links the uncoordinated water mol­ecule to the carboxyl groups of the complex. The dihedral angles between the carboxyl groups and the adjacent benzene rings are 10.28 (11) and 21.24 (9)°, while the two benzene rings and the two pyridine rings are oriented at dihedral angles of 6.90 (4) and 88.64 (4)°, respectively. In the crystal structure, O—H⋯O and N—H⋯O hydrogen bonds link the mol­ecules into a supra­molecular structure. A π–π contact between the benzene rings [centroid–centroid distance = 3.911 (1) Å] may further stabilize the crystal structure. Weak C—H⋯π inter­actions involving the pyridine rings also occur in the crystal structure.

## Related literature

For niacin, see: Krishnamachari (1974 ▶) and for the nicotinic acid derivative *N*,*N*-diethyl­nicotinamide, see: Bigoli *et al.* (1972 ▶). For related structures, see: Greenaway *et al.* (1984 ▶); Hökelek & Necefoğlu (1996 ▶); Hökelek *et al.* (2009*a* ▶,*b* ▶,*c* ▶,*d* ▶).
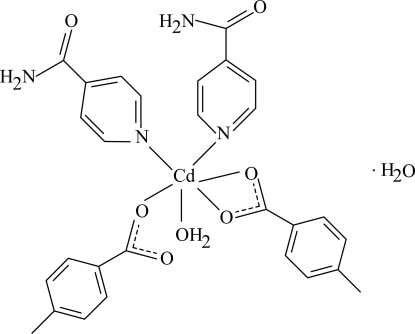


## Experimental

### 

#### Crystal data

[Cd(C_8_H_7_O_2_)_2_(C_6_H_6_N_2_O)_2_(H_2_O)]·H_2_O
*M* *_r_* = 662.97
Triclinic, 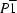

*a* = 9.5032 (2) Å
*b* = 12.3543 (3) Å
*c* = 13.6134 (3) Å
α = 78.278 (3)°
β = 69.776 (2)°
γ = 71.746 (3)°
*V* = 1416.18 (6) Å^3^
*Z* = 2
Mo *K*α radiation
μ = 0.83 mm^−1^
*T* = 102 K
0.40 × 0.20 × 0.15 mm

#### Data collection

Bruker Kappa APEXII CCD area-detector diffractometer
Absorption correction: multi-scan (*SADABS*; Bruker, 2005 ▶) *T*_min_ = 0.819, *T*_max_ = 0.881
25922 measured reflections
7154 independent reflections
6962 reflections with *I* > 2σ(*I*)
*R*_int_ = 0.021

#### Refinement

*R*[*F* ^2^ > 2σ(*F* ^2^)] = 0.020
*wR*(*F* ^2^) = 0.053
*S* = 1.14
7154 reflections
404 parameters
H atoms treated by a mixture of independent and constrained refinement
Δρ_max_ = 0.56 e Å^−3^
Δρ_min_ = −0.38 e Å^−3^

### 

Data collection: *APEX2* (Bruker, 2007 ▶); cell refinement: *SAINT* (Bruker, 2007 ▶); data reduction: *SAINT*; program(s) used to solve structure: *SHELXS97* (Sheldrick, 2008 ▶); program(s) used to refine structure: *SHELXL97* (Sheldrick, 2008 ▶); molecular graphics: *ORTEP-3 for Windows* (Farrugia, 1997 ▶); software used to prepare material for publication: *WinGX* (Farrugia, 1999 ▶) and *PLATON* (Spek, 2009 ▶).

## Supplementary Material

Crystal structure: contains datablocks I, global. DOI: 10.1107/S1600536810008366/xu2732sup1.cif

Structure factors: contains datablocks I. DOI: 10.1107/S1600536810008366/xu2732Isup2.hkl

Additional supplementary materials:  crystallographic information; 3D view; checkCIF report

## Figures and Tables

**Table 1:** Selected bond lengths (Å)

Cd1—O1	2.2478 (11)
Cd1—O3	2.4263 (11)
Cd1—O4	2.3794 (11)
Cd1—O7	2.2947 (11)
Cd1—N1	2.3295 (12)
Cd1—N3	2.3671 (13)

**Table 2:** Hydrogen-bond geometry (Å, °) *Cg*3 and *Cg*4 are the centroids of the N1/C17-C21 and N3/C23-C27 rings, respectively.

*D*—H⋯*A*	*D*—H	H⋯*A*	*D*⋯*A*	*D*—H⋯*A*
N2—H21⋯O5^i^	0.85 (2)	2.05 (2)	2.8990 (19)	177 (2)
N2—H22⋯O6^ii^	0.87 (3)	2.10 (3)	2.948 (2)	163 (2)
N4—H41⋯O8^iii^	0.87 (2)	1.99 (2)	2.822 (2)	160 (2)
N4—H42⋯O6^iv^	0.86 (2)	2.05 (2)	2.8979 (18)	171 (2)
O7—H71⋯O2^v^	0.79 (3)	1.93 (3)	2.7186 (19)	175 (2)
O7—H72⋯O3^ii^	0.80 (3)	1.97 (3)	2.7690 (18)	174 (3)
O8—H81⋯O4	0.79 (3)	2.21 (3)	2.8767 (18)	143 (3)
O8—H82⋯O1	0.80 (3)	1.93 (3)	2.7269 (18)	169 (3)
C6—H6⋯*Cg*4^vi^	0.93	2.82	3.720 (2)	163
C14—H14⋯*Cg*3^vii^	0.93	2.78	3.6840 (19)	164

Symmetry codes: (i) 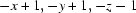; (ii) 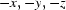; (iii) 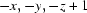; (iv) 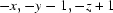; (v) 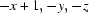; (vi) 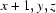; (vii) 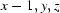.

**Fig. 1.**
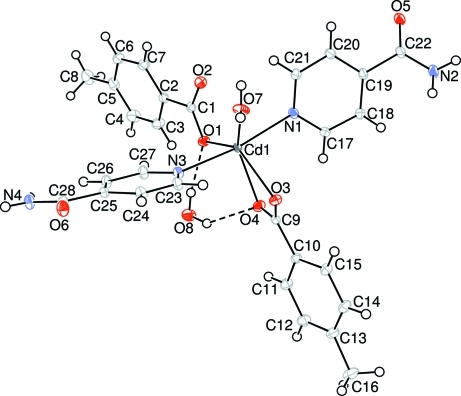
The molecular structure of the title molecule with the atom-numbering scheme. Displacement ellipsoids are drawn at the 30% probability level. Dashed lines indicate the hydrogen-bondings.

## supplementary crystallographic information

### Comment

As a part of our ongoing investigation on transition metal complexes of nicotinamide (NA), one form of niacin (Krishnamachari, 1974), and/or the nicotinic acid derivative *N*,*N*-diethylnicotinamide (DENA), an important respiratory stimulant (Bigoli *et al.*, 1972), the title compound was synthesized and its crystal structure is reported herein.

The title compound, (I), is a monomeric complex, where the Cd^II^ ion is surrounded by two 4-methylbenzoate (PMB) and two isonicotinamide (INA) ligands and one water molecule. One of the PMB ions acts as a bidentate ligand, while the other PMB and two INA are monodentate ligands. The crystal structures of similar complexes of Cd^II^, Co^II^, Mn^II^ and Zn^II^ ions, [Cd(C_8_H_5_O_3_)_2_(C_6_H_6_N_2_O)_2_(H_2_O)].H_2_O, (II) (Hökelek *et al.*, 2009a), [Co(C_9_H_10_NO_2_)_2_(C_6_H_6_N_2_O)(H_2_O)_2_], (III) (Hökelek *et al.*, 2009b), [Mn(C_9_H_10_NO_2_)_2_(C_6_H_6_N_2_O)(H_2_O)_2_], (IV) (Hökelek *et al.*, 2009c), [Zn_2_(DENA)_2_(C_7_H_5_O_3_)_4_].2H_2_O, (V) (Hökelek & Necefoğlu, 1996) and [Zn(C_8_H_8_NO_2_)_2_(C_6_H_6_N_2_O)_2_].H_2_O, (VI) (Hökelek *et al.*, 2009d) have also been reported. In (II), the two benzoate ions are coordinated to the Cd atom as bidentate ligands. In the other structures one of the benzoate ligands acts as a bidentate ligand, while the other is monodentate ligand.

In the title compound (Fig. 1), the average Cd—O bond length (Table 1) is 2.3371 (11) Å and the Cd atom is displaced out of the least-squares planes of the carboxylate groups (O1/C1/O2) and (O3/C9/O4) by 0.2697 (1) Å and 0.0105 (1) Å, respectively. The dihedral angle between the planar carboxylate groups and the adjacent benzene rings A (C2—C7) and B (C10—C15) are 10.28 (11)° and 21.24 (9)°, respectively, while those between rings A, B, C (N1/C17—C21) and D (N3/C23—C27) are A/B = 6.90 (4), A/C = 63.11 (5), A/D = 76.86 (4), B/C = 62.69 (5), B/D = 83.75 (4) and C/D = 88.64 (4) °. The intramolecular O—H···O hydrogen bonds (Table 2) link the water molecules to the carboxylate groups (O1/C1/O2) and (O3/C9/O4). In (I), the O3—Cd1—O4 angle is 54.71 (4)°. The corresponding O—M—O (where M is a metal) angles are 52.91 (4)° and 53.96 (4)° in (II), 60.70 (4)° in (III), 58.45 (9)° in (IV), 58.3 (3)° in (V), 60.03 (6)° in (VI) and 55.2 (1)° in [Cu(Asp)_2_(py)_2_] (where Asp is acetylsalicylate and py is pyridine) [(VII); Greenaway *et al.*, 1984].

In the crystal structure, intramolecular O—H···O and intermolecular O—H···O and N—H···O hydrogen bonds (Table 2) link the molecules into a supramolecular structure, in which they may be effective in the stabilization of the structure. The π–π contact between the benzene rings, Cg1—Cg2^i^, [symmetry code (i): 1 + x, y, z, where Cg1 and Cg2 are the centroids of rings A (C2—C7) and B (C10—C15)] may further stabilize the structure, with centroid-centroid distance of 3.911 (1) Å. There also exists two weak C—H···π interactions involving the pyridine rings C and D (Table 2).

### Experimental

The title compound was prepared by the reaction of 3CdSO_4_.8H_2_O (1.29 g, 5 mmol) in H_2_O (40 ml) and INA (1.22 g, 10 mmol) in H_2_O (15 ml) with sodium 4-methylbenzoate (1.58 g, 10 mmol) in H_2_O (350 ml). The mixture was filtered and set aside to crystallize at ambient temperature for one week, giving colorless single crystals.

### Refinement

Atoms H21, H22, H41, H42 (for NH_2_) and H71, H72, H81, H82 (for H_2_O) were located in a difference Fourier map and refined isotropically. The remaining H atoms were positioned geometrically with C—H = 0.93 and 0.96 Å for aromatic and methyl H atoms and constrained to ride on their parent atoms, with U_iso_(H) = xU_eq_(C), where x = 1.5 for methyl H and x = 1.2 for aromatic H atoms.

### Crystal data

**Table:**

[Cd(C_8_H_7_O_2_)_2_(C_6_H_6_N_2_O)_2_(H_2_O)]·H_2_O	*Z* = 2
*M**_r_* = 662.97	*F*(000) = 676
Triclinic, *P*1	*D*_x_ = 1.555 Mg m^−^^3^
Hall symbol: -P 1	Mo *K*α radiation, λ = 0.71073 Å
*a* = 9.5032 (2) Å	Cell parameters from 8576 reflections
*b* = 12.3543 (3) Å	θ = 2.4–28.3°
*c* = 13.6134 (3) Å	µ = 0.83 mm^−^^1^
α = 78.278 (3)°	*T* = 102 K
β = 69.776 (2)°	Block, colorless
γ = 71.746 (3)°	0.40 × 0.20 × 0.15 mm
*V* = 1416.18 (6) Å^3^	

### Data collection

**Table:**

Bruker Kappa APEXII CCD area-detector diffractometer	7154 independent reflections
Radiation source: fine-focus sealed tube	6962 reflections with *I* > 2σ(*I*)
graphite	*R*_int_ = 0.021
φ and ω scans	θ_max_ = 28.6°, θ_min_ = 1.6°
Absorption correction: multi-scan (*SADABS*; Bruker, 2005)	*h* = −12→12
*T*_min_ = 0.819, *T*_max_ = 0.881	*k* = −16→16
25922 measured reflections	*l* = −18→18

### Refinement

**Table:**

Refinement on *F*^2^	Primary atom site location: structure-invariant direct methods
Least-squares matrix: full	Secondary atom site location: difference Fourier map
*R*[*F*^2^ > 2σ(*F*^2^)] = 0.020	Hydrogen site location: inferred from neighbouring sites
*wR*(*F*^2^) = 0.053	H atoms treated by a mixture of independent and constrained refinement
*S* = 1.14	*w* = 1/[σ^2^(*F*_o_^2^) + (0.0175*P*)^2^ + 1.1925*P*] where *P* = (*F*_o_^2^ + 2*F*_c_^2^)/3
7154 reflections	(Δ/σ)_max_ = 0.001
404 parameters	Δρ_max_ = 0.56 e Å^−^^3^
0 restraints	Δρ_min_ = −0.38 e Å^−^^3^

### Special details

**Table:**

Geometry. All esds (except the esd in the dihedral angle between two l.s. planes) are estimated using the full covariance matrix. The cell esds are taken into account individually in the estimation of esds in distances, angles and torsion angles; correlations between esds in cell parameters are only used when they are defined by crystal symmetry. An approximate (isotropic) treatment of cell esds is used for estimating esds involving l.s. planes.
Refinement. Refinement of *F*^2^ against ALL reflections. The weighted *R*-factor wR and goodness of fit *S* are based on *F*^2^, conventional *R*-factors *R* are based on *F*, with *F* set to zero for negative *F*^2^. The threshold expression of *F*^2^ > σ(*F*^2^) is used only for calculating *R*-factors(gt) etc. and is not relevant to the choice of reflections for refinement. *R*-factors based on *F*^2^ are statistically about twice as large as those based on *F*, and *R*- factors based on ALL data will be even larger.

### Fractional atomic coordinates and isotropic or equivalent isotropic displacement parameters (Å^2^)

**Table:**

	*x*	*y*	*z*	*U*_iso_*/*U*_eq_	
Cd1	0.141608 (11)	0.121190 (8)	0.094078 (7)	0.00977 (3)	
O1	0.21408 (12)	0.17237 (9)	0.21480 (8)	0.0153 (2)	
O2	0.43486 (13)	0.06777 (9)	0.11883 (8)	0.0158 (2)	
O3	−0.09543 (12)	0.15690 (9)	0.04849 (8)	0.0150 (2)	
O4	−0.08901 (13)	0.26914 (10)	0.15327 (9)	0.0164 (2)	
O5	0.56683 (13)	0.41643 (10)	−0.38584 (9)	0.0199 (2)	
O6	−0.03527 (14)	−0.40999 (9)	0.37641 (9)	0.0178 (2)	
O7	0.25490 (14)	−0.02091 (10)	−0.01623 (9)	0.0183 (2)	
H71	0.345 (3)	−0.038 (2)	−0.0464 (19)	0.030 (6)*	
H72	0.214 (3)	−0.064 (2)	−0.0261 (18)	0.028 (6)*	
O8	−0.06722 (16)	0.23407 (12)	0.36356 (10)	0.0224 (2)	
H81	−0.115 (3)	0.252 (2)	0.323 (2)	0.039 (7)*	
H82	0.021 (3)	0.216 (2)	0.326 (2)	0.039 (7)*	
N1	0.24879 (15)	0.23682 (11)	−0.05300 (10)	0.0131 (2)	
N2	0.32591 (17)	0.46852 (14)	−0.40532 (11)	0.0222 (3)	
H21	0.355 (3)	0.502 (2)	−0.4672 (19)	0.029 (6)*	
H22	0.235 (3)	0.4542 (19)	−0.3832 (18)	0.027 (6)*	
N3	0.06838 (15)	−0.03017 (11)	0.21984 (10)	0.0139 (2)	
N4	0.02873 (16)	−0.36600 (12)	0.50561 (10)	0.0162 (2)	
H41	0.056 (2)	−0.3201 (18)	0.5322 (17)	0.022 (5)*	
H42	0.020 (2)	−0.4306 (19)	0.5415 (16)	0.019 (5)*	
C1	0.35814 (17)	0.12189 (12)	0.19750 (11)	0.0122 (3)	
C2	0.43118 (17)	0.12606 (13)	0.27780 (11)	0.0138 (3)	
C3	0.34802 (19)	0.19738 (14)	0.35725 (13)	0.0190 (3)	
H3	0.2493	0.2439	0.3587	0.023*	
C4	0.4114 (2)	0.19972 (16)	0.43464 (13)	0.0223 (3)	
H4	0.3544	0.2478	0.4874	0.027*	
C5	0.55876 (19)	0.13100 (15)	0.43413 (12)	0.0196 (3)	
C6	0.64170 (19)	0.06017 (15)	0.35394 (13)	0.0208 (3)	
H6	0.7406	0.0138	0.3523	0.025*	
C7	0.57917 (18)	0.05760 (14)	0.27640 (12)	0.0174 (3)	
H7	0.6364	0.0099	0.2233	0.021*	
C8	0.6266 (2)	0.13244 (18)	0.51874 (14)	0.0276 (4)	
H8A	0.7263	0.0775	0.5079	0.041*	
H8B	0.5582	0.1132	0.5865	0.041*	
H8C	0.6384	0.2075	0.5157	0.041*	
C9	−0.16048 (17)	0.24072 (12)	0.10406 (11)	0.0123 (3)	
C10	−0.32334 (17)	0.30742 (12)	0.10992 (12)	0.0130 (3)	
C11	−0.41385 (18)	0.37317 (13)	0.19319 (12)	0.0166 (3)	
H11	−0.3706	0.3785	0.2432	0.020*	
C12	−0.56823 (19)	0.43074 (14)	0.20192 (13)	0.0195 (3)	
H12	−0.6280	0.4732	0.2584	0.023*	
C13	−0.63418 (18)	0.42543 (13)	0.12678 (13)	0.0177 (3)	
C14	−0.54103 (18)	0.36423 (13)	0.04114 (13)	0.0176 (3)	
H14	−0.5822	0.3633	−0.0114	0.021*	
C15	−0.38809 (18)	0.30477 (13)	0.03297 (12)	0.0152 (3)	
H15	−0.3283	0.2629	−0.0240	0.018*	
C16	−0.80317 (19)	0.48229 (16)	0.13748 (15)	0.0257 (4)	
H16A	−0.8456	0.5324	0.1920	0.039*	
H16B	−0.8590	0.4248	0.1551	0.039*	
H16C	−0.8127	0.5259	0.0721	0.039*	
C17	0.16137 (17)	0.33676 (13)	−0.08530 (12)	0.0152 (3)	
H17	0.0612	0.3651	−0.0419	0.018*	
C18	0.21389 (18)	0.39975 (13)	−0.18067 (12)	0.0166 (3)	
H18	0.1500	0.4686	−0.2009	0.020*	
C19	0.36446 (17)	0.35759 (13)	−0.24544 (11)	0.0134 (3)	
C20	0.45742 (18)	0.25694 (13)	−0.20997 (12)	0.0153 (3)	
H20	0.5599	0.2288	−0.2501	0.018*	
C21	0.39564 (18)	0.19876 (13)	−0.11404 (12)	0.0148 (3)	
H21A	0.4582	0.1308	−0.0911	0.018*	
C22	0.42827 (18)	0.41740 (13)	−0.35244 (12)	0.0154 (3)	
C23	0.01222 (18)	−0.10290 (13)	0.19260 (12)	0.0153 (3)	
H23	−0.0121	−0.0849	0.1296	0.018*	
C24	−0.01121 (18)	−0.20371 (13)	0.25425 (12)	0.0158 (3)	
H24	−0.0495	−0.2523	0.2325	0.019*	
C25	0.02332 (17)	−0.23100 (12)	0.34907 (11)	0.0125 (3)	
C26	0.07689 (18)	−0.15443 (13)	0.37961 (12)	0.0160 (3)	
H26	0.0976	−0.1688	0.4437	0.019*	
C27	0.09879 (18)	−0.05607 (13)	0.31243 (12)	0.0156 (3)	
H27	0.1363	−0.0057	0.3326	0.019*	
C28	0.00343 (17)	−0.34324 (12)	0.41280 (11)	0.0131 (3)	

### Atomic displacement parameters (Å^2^)

**Table:**

	*U*^11^	*U*^22^	*U*^33^	*U*^12^	*U*^13^	*U*^23^
Cd1	0.01036 (5)	0.01012 (5)	0.00874 (5)	−0.00363 (4)	−0.00264 (4)	0.00016 (3)
O1	0.0118 (5)	0.0192 (5)	0.0162 (5)	−0.0038 (4)	−0.0054 (4)	−0.0029 (4)
O2	0.0165 (5)	0.0163 (5)	0.0152 (5)	−0.0032 (4)	−0.0054 (4)	−0.0038 (4)
O3	0.0122 (5)	0.0160 (5)	0.0169 (5)	−0.0018 (4)	−0.0048 (4)	−0.0040 (4)
O4	0.0142 (5)	0.0201 (5)	0.0177 (5)	−0.0041 (4)	−0.0076 (4)	−0.0034 (4)
O5	0.0164 (5)	0.0272 (6)	0.0148 (5)	−0.0095 (5)	−0.0039 (4)	0.0046 (4)
O6	0.0239 (6)	0.0157 (5)	0.0185 (5)	−0.0105 (4)	−0.0098 (4)	0.0023 (4)
O7	0.0141 (5)	0.0195 (6)	0.0213 (6)	−0.0072 (5)	0.0013 (4)	−0.0100 (4)
O8	0.0184 (6)	0.0347 (7)	0.0148 (5)	−0.0063 (5)	−0.0039 (5)	−0.0075 (5)
N1	0.0138 (6)	0.0132 (6)	0.0125 (6)	−0.0055 (5)	−0.0033 (5)	0.0003 (4)
N2	0.0179 (7)	0.0322 (8)	0.0158 (6)	−0.0125 (6)	−0.0065 (5)	0.0105 (6)
N3	0.0152 (6)	0.0144 (6)	0.0121 (6)	−0.0049 (5)	−0.0038 (5)	−0.0006 (4)
N4	0.0225 (7)	0.0131 (6)	0.0147 (6)	−0.0078 (5)	−0.0072 (5)	0.0024 (5)
C1	0.0128 (6)	0.0106 (6)	0.0138 (6)	−0.0055 (5)	−0.0045 (5)	0.0017 (5)
C2	0.0135 (6)	0.0162 (7)	0.0128 (6)	−0.0060 (5)	−0.0045 (5)	0.0001 (5)
C3	0.0157 (7)	0.0226 (8)	0.0205 (7)	−0.0035 (6)	−0.0068 (6)	−0.0063 (6)
C4	0.0224 (8)	0.0292 (9)	0.0190 (8)	−0.0074 (7)	−0.0063 (6)	−0.0091 (6)
C5	0.0207 (8)	0.0278 (8)	0.0155 (7)	−0.0132 (7)	−0.0078 (6)	0.0014 (6)
C6	0.0142 (7)	0.0299 (9)	0.0190 (7)	−0.0055 (6)	−0.0079 (6)	0.0006 (6)
C7	0.0146 (7)	0.0223 (8)	0.0145 (7)	−0.0037 (6)	−0.0044 (6)	−0.0022 (6)
C8	0.0299 (9)	0.0432 (11)	0.0195 (8)	−0.0202 (8)	−0.0128 (7)	0.0018 (7)
C9	0.0119 (6)	0.0136 (6)	0.0109 (6)	−0.0049 (5)	−0.0029 (5)	0.0016 (5)
C10	0.0114 (6)	0.0124 (6)	0.0157 (7)	−0.0038 (5)	−0.0049 (5)	−0.0002 (5)
C11	0.0158 (7)	0.0173 (7)	0.0183 (7)	−0.0032 (6)	−0.0067 (6)	−0.0042 (6)
C12	0.0162 (7)	0.0189 (7)	0.0209 (8)	−0.0012 (6)	−0.0037 (6)	−0.0057 (6)
C13	0.0130 (7)	0.0150 (7)	0.0246 (8)	−0.0029 (5)	−0.0067 (6)	−0.0004 (6)
C14	0.0169 (7)	0.0159 (7)	0.0241 (8)	−0.0038 (6)	−0.0119 (6)	−0.0019 (6)
C15	0.0151 (7)	0.0136 (7)	0.0177 (7)	−0.0028 (5)	−0.0064 (6)	−0.0027 (5)
C16	0.0142 (7)	0.0257 (9)	0.0352 (10)	0.0002 (6)	−0.0095 (7)	−0.0039 (7)
C17	0.0117 (6)	0.0170 (7)	0.0145 (7)	−0.0040 (5)	−0.0029 (5)	0.0015 (5)
C18	0.0143 (7)	0.0164 (7)	0.0162 (7)	−0.0037 (6)	−0.0051 (6)	0.0044 (6)
C19	0.0151 (7)	0.0151 (7)	0.0114 (6)	−0.0072 (5)	−0.0047 (5)	0.0018 (5)
C20	0.0143 (7)	0.0154 (7)	0.0136 (7)	−0.0040 (5)	−0.0017 (5)	−0.0008 (5)
C21	0.0154 (7)	0.0129 (7)	0.0144 (7)	−0.0029 (5)	−0.0042 (5)	0.0002 (5)
C22	0.0174 (7)	0.0161 (7)	0.0123 (6)	−0.0073 (6)	−0.0035 (5)	0.0019 (5)
C23	0.0190 (7)	0.0170 (7)	0.0115 (6)	−0.0071 (6)	−0.0059 (5)	0.0009 (5)
C24	0.0216 (7)	0.0152 (7)	0.0132 (7)	−0.0082 (6)	−0.0058 (6)	−0.0012 (5)
C25	0.0124 (6)	0.0117 (6)	0.0123 (6)	−0.0037 (5)	−0.0033 (5)	0.0012 (5)
C26	0.0202 (7)	0.0171 (7)	0.0142 (7)	−0.0088 (6)	−0.0088 (6)	0.0028 (5)
C27	0.0203 (7)	0.0154 (7)	0.0145 (7)	−0.0091 (6)	−0.0073 (6)	0.0014 (5)
C28	0.0111 (6)	0.0128 (6)	0.0136 (6)	−0.0036 (5)	−0.0025 (5)	0.0009 (5)

### Geometric parameters (Å, °)

**Table:**

Cd1—O1	2.2478 (11)	C8—C5	1.507 (2)
Cd1—O3	2.4263 (11)	C8—H8A	0.9600
Cd1—O4	2.3794 (11)	C8—H8B	0.9600
Cd1—O7	2.2947 (11)	C8—H8C	0.9600
Cd1—N1	2.3295 (12)	C9—C10	1.491 (2)
Cd1—N3	2.3671 (13)	C10—C11	1.395 (2)
O1—C1	1.2730 (18)	C10—C15	1.396 (2)
O2—C1	1.2535 (18)	C11—H11	0.9300
O3—C9	1.2730 (18)	C12—C11	1.390 (2)
O4—C9	1.2622 (18)	C12—C13	1.393 (2)
O5—C22	1.2325 (19)	C12—H12	0.9300
O6—C28	1.2422 (18)	C13—C16	1.506 (2)
O7—H71	0.79 (3)	C14—C13	1.393 (2)
O7—H72	0.80 (2)	C14—C15	1.385 (2)
O8—H81	0.79 (3)	C14—H14	0.9300
O8—H82	0.81 (3)	C15—H15	0.9300
N1—C17	1.3406 (19)	C16—H16A	0.9600
N1—C21	1.3435 (19)	C16—H16B	0.9600
N2—C22	1.333 (2)	C16—H16C	0.9600
N2—H21	0.85 (2)	C17—H17	0.9300
N2—H22	0.87 (2)	C18—C17	1.387 (2)
N3—C23	1.3425 (19)	C18—C19	1.394 (2)
N3—C27	1.3433 (19)	C18—H18	0.9300
N4—C28	1.325 (2)	C20—C19	1.387 (2)
N4—H41	0.87 (2)	C20—H20	0.9300
N4—H42	0.86 (2)	C21—C20	1.385 (2)
C1—C2	1.500 (2)	C21—H21A	0.9300
C2—C3	1.390 (2)	C22—C19	1.506 (2)
C2—C7	1.392 (2)	C23—C24	1.387 (2)
C3—C4	1.391 (2)	C23—H23	0.9300
C3—H3	0.9300	C24—H24	0.9300
C4—C5	1.391 (2)	C25—C24	1.391 (2)
C4—H4	0.9300	C26—C25	1.391 (2)
C6—C5	1.393 (2)	C26—C27	1.390 (2)
C6—C7	1.388 (2)	C26—H26	0.9300
C6—H6	0.9300	C27—H27	0.9300
C7—H7	0.9300	C28—C25	1.5077 (19)
			
O1—Cd1—O3	137.23 (4)	O4—C9—O3	121.19 (13)
O1—Cd1—O4	83.65 (4)	O4—C9—C10	119.45 (13)
O1—Cd1—O7	133.14 (4)	C11—C10—C15	118.94 (14)
O1—Cd1—N1	99.39 (4)	C11—C10—C9	120.24 (13)
O1—Cd1—N3	87.65 (4)	C15—C10—C9	120.82 (13)
O4—Cd1—O3	54.71 (4)	C10—C11—H11	119.8
O7—Cd1—O3	88.51 (4)	C12—C11—C10	120.44 (14)
O7—Cd1—O4	143.07 (4)	C12—C11—H11	119.8
O7—Cd1—N1	84.40 (4)	C11—C12—C13	120.59 (15)
O7—Cd1—N3	82.88 (4)	C11—C12—H12	119.7
N1—Cd1—O3	93.12 (4)	C13—C12—H12	119.7
N1—Cd1—O4	93.53 (4)	C12—C13—C14	118.64 (14)
N1—Cd1—N3	167.06 (4)	C12—C13—C16	121.48 (15)
N3—Cd1—O3	88.91 (4)	C14—C13—C16	119.87 (15)
N3—Cd1—O4	98.06 (4)	C13—C14—H14	119.5
C1—O1—Cd1	105.84 (9)	C15—C14—C13	121.06 (14)
C9—O3—Cd1	90.83 (8)	C15—C14—H14	119.5
C9—O4—Cd1	93.27 (9)	C10—C15—H15	119.9
Cd1—O7—H71	123.1 (17)	C14—C15—C10	120.21 (14)
Cd1—O7—H72	127.0 (17)	C14—C15—H15	119.9
H72—O7—H71	110 (2)	C13—C16—H16A	109.5
H81—O8—H82	103 (3)	C13—C16—H16B	109.5
C17—N1—Cd1	121.04 (10)	C13—C16—H16C	109.5
C17—N1—C21	118.25 (13)	H16A—C16—H16B	109.5
C21—N1—Cd1	120.31 (10)	H16A—C16—H16C	109.5
C22—N2—H21	120.3 (15)	H16B—C16—H16C	109.5
C22—N2—H22	119.5 (15)	N1—C17—C18	122.87 (14)
H22—N2—H21	119 (2)	N1—C17—H17	118.6
C23—N3—Cd1	118.71 (10)	C18—C17—H17	118.6
C23—N3—C27	117.68 (13)	C17—C18—C19	118.55 (14)
C27—N3—Cd1	123.03 (10)	C17—C18—H18	120.7
C28—N4—H41	124.1 (14)	C19—C18—H18	120.7
C28—N4—H42	118.4 (14)	C18—C19—C22	122.10 (14)
H42—N4—H41	117.4 (19)	C20—C19—C18	118.62 (13)
O1—C1—C2	117.01 (13)	C20—C19—C22	119.28 (13)
O2—C1—O1	121.75 (13)	C19—C20—H20	120.4
O2—C1—C2	121.20 (13)	C21—C20—C19	119.15 (14)
C3—C2—C1	119.49 (14)	C21—C20—H20	120.4
C3—C2—C7	119.02 (14)	N1—C21—C20	122.46 (14)
C7—C2—C1	121.47 (13)	N1—C21—H21A	118.8
C2—C3—C4	120.48 (15)	C20—C21—H21A	118.8
C2—C3—H3	119.8	O5—C22—N2	124.40 (14)
C4—C3—H3	119.8	O5—C22—C19	120.11 (14)
C3—C4—H4	119.6	N2—C22—C19	115.49 (13)
C5—C4—C3	120.82 (15)	N3—C23—C24	122.90 (14)
C5—C4—H4	119.6	N3—C23—H23	118.5
C4—C5—C6	118.36 (14)	C24—C23—H23	118.5
C4—C5—C8	120.82 (16)	C23—C24—C25	119.07 (14)
C6—C5—C8	120.82 (16)	C23—C24—H24	120.5
C5—C6—H6	119.5	C25—C24—H24	120.5
C7—C6—C5	121.08 (15)	C24—C25—C26	118.47 (13)
C7—C6—H6	119.5	C24—C25—C28	118.30 (13)
C2—C7—H7	119.9	C26—C25—C28	123.23 (13)
C6—C7—C2	120.24 (15)	C25—C26—H26	120.7
C6—C7—H7	119.9	C27—C26—C25	118.64 (14)
C5—C8—H8A	109.5	C27—C26—H26	120.7
C5—C8—H8B	109.5	N3—C27—C26	123.20 (14)
C5—C8—H8C	109.5	N3—C27—H27	118.4
H8A—C8—H8B	109.5	C26—C27—H27	118.4
H8A—C8—H8C	109.5	O6—C28—N4	123.03 (14)
H8B—C8—H8C	109.5	O6—C28—C25	119.00 (13)
O3—C9—C10	119.35 (13)	N4—C28—C25	117.97 (13)
			
O3—Cd1—O1—C1	178.82 (8)	O1—C1—C2—C3	−9.6 (2)
O4—Cd1—O1—C1	−168.65 (9)	O1—C1—C2—C7	168.54 (14)
O7—Cd1—O1—C1	15.00 (11)	O2—C1—C2—C3	172.66 (14)
N1—Cd1—O1—C1	−76.11 (9)	O2—C1—C2—C7	−9.2 (2)
N3—Cd1—O1—C1	92.97 (9)	C1—C2—C3—C4	177.80 (14)
O1—Cd1—O3—C9	15.18 (11)	C7—C2—C3—C4	−0.4 (2)
O4—Cd1—O3—C9	−0.14 (8)	C1—C2—C7—C6	−177.67 (14)
O7—Cd1—O3—C9	−176.56 (9)	C3—C2—C7—C6	0.5 (2)
N1—Cd1—O3—C9	−92.25 (9)	C2—C3—C4—C5	0.0 (3)
N3—Cd1—O3—C9	100.53 (9)	C3—C4—C5—C6	0.3 (3)
O1—Cd1—O4—C9	−169.47 (9)	C3—C4—C5—C8	−179.23 (16)
O3—Cd1—O4—C9	0.14 (8)	C5—C6—C7—C2	−0.2 (2)
O7—Cd1—O4—C9	6.09 (12)	C7—C6—C5—C4	−0.2 (2)
N1—Cd1—O4—C9	91.47 (9)	C7—C6—C5—C8	179.32 (15)
N3—Cd1—O4—C9	−82.76 (9)	O3—C9—C10—C11	159.36 (14)
O1—Cd1—N1—C17	−98.58 (11)	O3—C9—C10—C15	−20.3 (2)
O1—Cd1—N1—C21	88.82 (11)	O4—C9—C10—C11	−21.4 (2)
O3—Cd1—N1—C17	40.37 (11)	O4—C9—C10—C15	158.92 (14)
O3—Cd1—N1—C21	−132.23 (11)	C9—C10—C11—C12	−176.66 (14)
O4—Cd1—N1—C17	−14.44 (11)	C15—C10—C11—C12	3.0 (2)
O4—Cd1—N1—C21	172.96 (11)	C9—C10—C15—C14	177.96 (14)
O7—Cd1—N1—C17	128.57 (12)	C11—C10—C15—C14	−1.7 (2)
O7—Cd1—N1—C21	−44.04 (11)	C13—C12—C11—C10	−1.2 (2)
N3—Cd1—N1—C17	139.16 (18)	C11—C12—C13—C14	−1.9 (2)
N3—Cd1—N1—C21	−33.4 (3)	C11—C12—C13—C16	176.95 (15)
O1—Cd1—N3—C23	−178.51 (11)	C15—C14—C13—C12	3.3 (2)
O1—Cd1—N3—C27	−7.44 (12)	C15—C14—C13—C16	−175.65 (15)
O3—Cd1—N3—C23	44.13 (11)	C13—C14—C15—C10	−1.5 (2)
O3—Cd1—N3—C27	−144.80 (12)	C19—C18—C17—N1	−0.4 (2)
O4—Cd1—N3—C23	98.24 (11)	C17—C18—C19—C20	−2.6 (2)
O4—Cd1—N3—C27	−90.69 (12)	C17—C18—C19—C22	177.02 (14)
O7—Cd1—N3—C23	−44.50 (11)	C21—C20—C19—C18	3.1 (2)
O7—Cd1—N3—C27	126.57 (12)	C21—C20—C19—C22	−176.49 (13)
N1—Cd1—N3—C23	−55.1 (2)	N1—C21—C20—C19	−0.8 (2)
N1—Cd1—N3—C27	115.9 (2)	O5—C22—C19—C18	145.44 (16)
Cd1—N1—C17—C18	−170.02 (12)	O5—C22—C19—C20	−35.0 (2)
C21—N1—C17—C18	2.7 (2)	N2—C22—C19—C18	−34.9 (2)
Cd1—N1—C21—C20	170.65 (11)	N2—C22—C19—C20	144.70 (15)
C17—N1—C21—C20	−2.2 (2)	N3—C23—C24—C25	0.6 (2)
Cd1—N3—C23—C24	169.79 (12)	C26—C25—C24—C23	1.5 (2)
C27—N3—C23—C24	−1.8 (2)	C28—C25—C24—C23	−177.50 (14)
Cd1—N3—C27—C26	−170.25 (12)	C27—C26—C25—C24	−2.2 (2)
C23—N3—C27—C26	0.9 (2)	C27—C26—C25—C28	176.66 (14)
Cd1—O1—C1—O2	7.16 (16)	C25—C26—C27—N3	1.1 (2)
Cd1—O1—C1—C2	−170.52 (10)	O6—C28—C25—C24	3.6 (2)
Cd1—O3—C9—O4	0.24 (14)	O6—C28—C25—C26	−175.30 (14)
Cd1—O3—C9—C10	179.43 (11)	N4—C28—C25—C24	−176.55 (14)
Cd1—O4—C9—O3	−0.25 (14)	N4—C28—C25—C26	4.5 (2)
Cd1—O4—C9—C10	−179.43 (11)		

### Hydrogen-bond geometry (Å, °)

**Table:**

Cg3 and Cg4 are the centroids of the N1/C17-C21 and N3/C23-C27 rings, respectively.

**Table:**

*D*—H···*A*	*D*—H	H···*A*	*D*···*A*	*D*—H···*A*
N2—H21···O5^i^	0.85 (2)	2.05 (2)	2.8990 (19)	177 (2)
N2—H22···O6^ii^	0.87 (3)	2.10 (3)	2.948 (2)	163 (2)
N4—H41···O8^iii^	0.87 (2)	1.99 (2)	2.822 (2)	160 (2)
N4—H42···O6^iv^	0.86 (2)	2.05 (2)	2.8979 (18)	171 (2)
O7—H71···O2^v^	0.79 (3)	1.93 (3)	2.7186 (19)	175 (2)
O7—H72···O3^ii^	0.80 (3)	1.97 (3)	2.7690 (18)	174 (3)
O8—H81···O4	0.79 (3)	2.21 (3)	2.8767 (18)	143 (3)
O8—H82···O1	0.80 (3)	1.93 (3)	2.7269 (18)	169 (3)
C6—H6···Cg4^vi^	0.93	2.82	3.7196 (20)	163
C14—H14···Cg3^vii^	0.93	2.78	3.6840 (19)	164

Symmetry codes: (i) −*x*+1, −*y*+1, −*z*−1; (ii) −*x*, −*y*, −*z*; (iii) −*x*, −*y*, −*z*+1; (iv) −*x*, −*y*−1, −*z*+1; (v) −*x*+1, −*y*, −*z*; (vi) *x*+1, *y*, *z*; (vii) *x*−1, *y*, *z*.
